# Gene variations in Autism Spectrum Disorder are associated with alternation of gut microbiota, metabolites and cytokines

**DOI:** 10.1080/19490976.2020.1854967

**Published:** 2021-01-08

**Authors:** Zhi Liu, Xuhua Mao, Zhou Dan, Yang Pei, Rui Xu, Mengchen Guo, Kangjian Liu, Faming Zhang, Junyu Chen, Chuan Su, Yaoyao Zhuang, Junming Tang, Yankai Xia, Lianhong Qin, Zhibin Hu, Xingyin Liu

**Affiliations:** a State Key Laboratory of Reproductive Medicine, Center of Global Health, Nanjing Medical University, Nanjing, China; b Key Laboratory of Pathogen Biology of Jiangsu Province, Department of Pathogen Biology, Nanjing Medical University, Nanjing, China; c Key Laboratory of Human Functional Genomics of Jiangsu Province, Nanjing Medical University, Nanjing, China; d Department of Clinical Laboratory, Affiliated Yixing People’s Hospital, Jiangsu University, Wuxi, China; e Key Laboratory of Holistic Integrative Enterology, Second Affiliated Hospital of Nanjing Medical University, Najing, China; f Children Growth Center of Bo’ai Homestead in Yixing, Yixing, China

**Keywords:** Autism, genetic variation, gut microbiota, metabolites, cytokine

## Abstract

The genetic variations and dysbiosis of gut microbiota are associated with ASD. However, the role of the microbiota in the etiology of ASD in terms of host genetic susceptibility remains unclear. This study aims to systematically explore the interplay between host genetic variation and gut microbiota in ASD children. Whole-exon sequencing was applied to 26 ASD children and 26 matched controls to identify the single nucleotide variations (SNVs) in ASD. Our previous study revealed alteration in gut microbiota and disorder of metabolism activity in ASD for this cohort. Systematic bioinformatic analyses were further performed to identify associations between SNVs and gut microbiota, as well as their metabolites. The ASD SNVs were significantly enriched in genes associated with innate immune response, protein glycosylation process, and retrograde axonal transport. These SNVs were also correlated with the microbiome composition and a broad aspect of microbial functions, especially metabolism. Additionally, the abundance of metabolites involved in the metabolic network of neurotransmitters was inferred to be causally related to specific SNVs and microbes. Furthermore, our data suggested that the interaction of host genetics and gut microbes may play a crucial role in the immune and metabolism homeostasis of ASD. This study may provide valuable clues to investigate the interaction of host genetic variations and gut microbiota in the pathogenesis of ASD.

## Introduction

Autism Spectrum Disorder (ASD) has genetic risk factors as there is a much higher concordance rate for the disease in monozygotic twins than indizygotic ones^1^. Previous large-cohort studies have revealed a large set of polymorphisms conferring various levels of risk.^2–4^ However, the de novo mutations, common variants, and short nucleotide polymorphisms identified across numerous ASD cases altogether only account for approximately 50% of the cases.^5,6^ As such, many studies highlight the possibility of environmental risk factors and associated medical co-morbidities that may contribute to the core neurobehavioral symptoms of the disorder.^7,8^

ASD children suffer from a range of gastrointestinal (GI) symptoms, such as gaseousness, diarrhea, and constipation, with the prevalence shown to be anywhere from 9% to 91%.^9^ These associations of ASD with a greater prevalence of GI symptoms motivate explorations of the role of gut microbiome in ASD pathogenesis, which is emerging as a key regulator of intestinal physiology, neuroimmunity, and host behavior. Growing studies have reported dysbiosis of the gut microbiota in ASD individuals. For example, studies have observed an altered abundance of *Sutterella, Desulfovibrio, Bacteroides vulgatus, Akkermansia, Prevotella, Coprococcus*, and Firmicutes in ASD patients.^10,11^ The gnotobiotic animal and probiotic studies demonstrated that alteration of microbiota can directly cause behavioral abnormalities and neuropathological endophenotypes of ASD. The phenotype can be improved by transplantation of normal gut microbiome.^12^

Microbial communities influence human physiology through their metabolites, cellular and molecular components and provide crucial signals for the development and function of the immune system.^13,14^ Both metabolites and immunity disorders were observed in ASD children. In the ASD mice model, 5-aminovaleric acid (5AV) was significantly lower than in the normal controls (TD), and the administration of 5AV improved behaviors in ASD mice.^15^ Elevated levels of IL-17α, IL-4, and IL-10 have been detected in the serum of a subset of autistic children.^16–18^

Recent studies have shown the role of host genetics in shaping both the overall microbiome composition and the individual bacterial taxa. For instance, Knight *et al*.^19^ reported that Crohn’s risk variants located in the NOD2 gene were associated with changes in the abundance of Enterobacteriaceae. A genome-wide host genetics and microbiome association conducted in HMP healthy cohort validated the associations between a loss-of-function variant in the fucosyltransferase 2 (*FUT2*) and a variant conferring hypolactasia near the lactase *(LCT) g*ene, with *Bifidobacterium longum* abundance in the stool.^20^ Also, our previous study has found that the genetic variation of the Jmjc domain of lysine demethylase 5 (*KDM5*) protein in drosophila can lead to compositional changes in the gut microbiota, overactivation of innate immunity pathway, and abnormal level of 5-HT, then the autism-like behaviors.^21^

Nevertheless, the detailed association study of host genetic variation, gut microbiota-metabolites, the immune system in ASD remains unknown. In this study, to investigate the nature and extent of host genome-microbiome interplay in ASD, a total of 26 ASD patients with constipation and 26 matched typically developed children (TD) were supplied to exon sequencing. The SNVs enriched in ASD were identified by comparing with the TDs, and removing SNVs with minor allele frequency (MAF) more than 5% as recorded in ExAc, 1000 Genome database, and the Chinese Millionome Database (CMDB). Then the fecal microbiome, fecal metabolites, and serum cytokines were characterized to dissect the association with the enriched SNVs. Besides, the causal relationship between host SNVs and gut microbiota was inferred. The association analysis of functional genetic variations, gut microbiota, metabolites, and cytokines can provide novel and insightful clues revealing the pathogenesis of ASD diseases.

## Results

### SNVs in ASD are related to the gut microbiome

**Figure 1.**
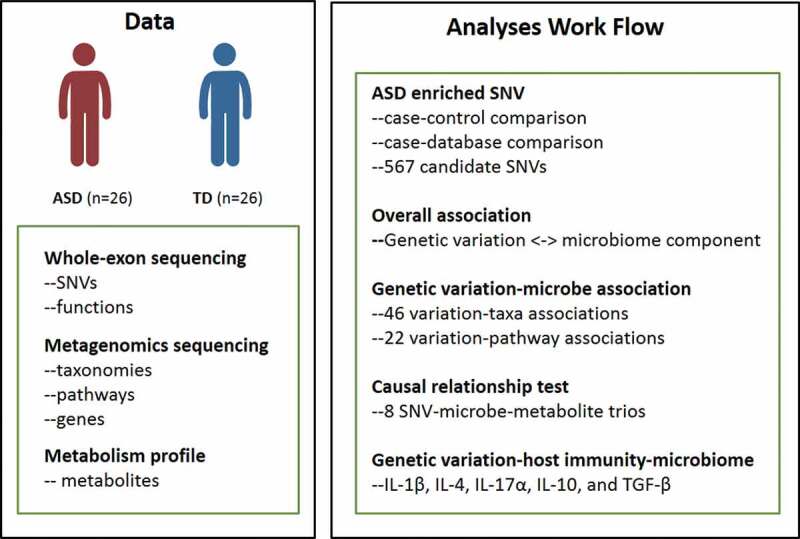
Schematic overview of the study

**Figure 2.**
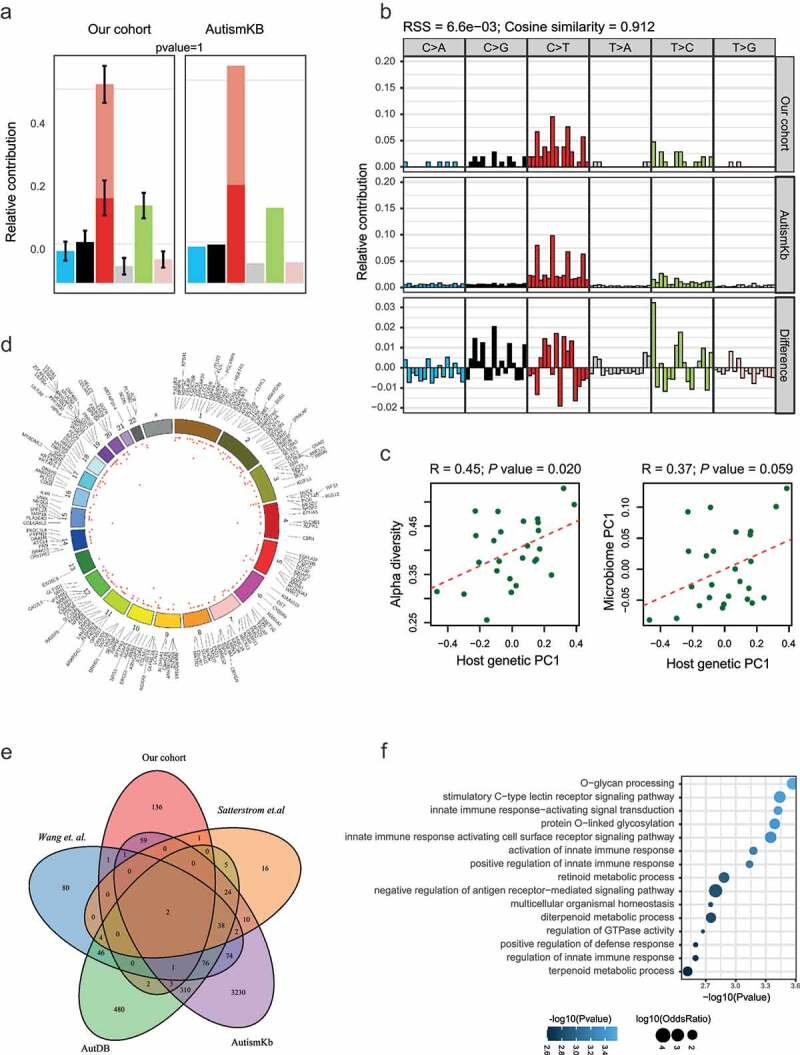
SNVs enriched in ASD children. (A-B) The comparison of the mutation pattern (A) and mutation signature(B) between our identified SNVs and mutations collected from the AutismKb database. (C) Correlation of the first PC of host genetic data (x-axis) and alpha diversity (left panel) or first PC of the stool microbiome (right panel). (D) Circos plot of the functional SNVs enriched in ASD. The height of the points represents the number of mutant samples. Genes mapped by SNVs were labeled outside the circle. (E) Overlap of genes with the ASD enriched SNVs in our studies with those collected from other studies. (F) Functional annotation of genes with functional SNVs

A total of 567 exonic SNVs (Table S1) were identified in our ASD cohort using the matched TD cohort and the public databases as control (Figure 1). These SNVs shared the same mutation pattern (Figure 2(a)) and mutation signature (Figure 2(b)) with the previously reported ASD-related mutations collected in the AutismKB, a knowledgebase for the genetic evidence of autism spectrum disorder.^22^ We calculated the coordinates underlying variability in the host SNV genotype data using Principal Component Analysis (PCA). We then computed alpha diversity, a measure of within-sample microbial diversity (Simpson index within a sample). We found that the first coordinated of the functional SNVs (protein-altering SNVs, including non-synonymous, stop-gain, and stop-loss SNVs) was significantly correlated with alpha diversity (Figure 2(c), R = 0.45, *P* = .020) of the gut microbiome. The altered alpha diversity links to lots of human health disease,^23,24^ including autism.^15^ Our results suggested a possible role for host genetic variations in shaping it. Next, we looked for the association of host genetics with the overall composition of the gut microbiome. A marginally significant correlation was observed between the first principal coordinate of functional SNVs and microbiome PC1 (Figure 2(c), R = 0.37, *P* = .059). However, when considering the total enriched SNVs, no significant correlation between the host genetics and microbiome diversity or composition was detected (Figure S1). These correlations suggest a potential relationship between the functional SNVs and microbiome.

We performed whole exome sequencing of the host genome, whole genome shotgun sequencing and metabolite profile of fecal samples of 26 ASD individuals and 26 TDs. Associations were tested between genetic variations and microbes, as well as microbial genes, metabolites and pathways, and host immunity.

The functional SNVs were mapped to 206 genes (Figure 2(d)), including 70 previously reported genes,^4,22,25,26^ such as *MUC4, SETD5*, and *ANKRD11*^27-29^ (Figure 2(e)). Functional enrichment analysis on these genes revealed a significant enrichment on the protein glycosylation process, innate immune response, and retinoid metabolic process (figure 2(f)). The mucins encoded by MUC family genes are O-glycosylated proteins that play an essential role in forming protective mucous barriers on epithelial surfaces,^30^ and mucins are components of innate and adaptive immune responses to mucosal infection.^31^ Increasing evidence has shown that variants in genes encoding glycosylated extracellular proteins or enzymes (glycogene) involved in glycosylation may contribute to the etiology and pathogenesis of ASD.^32^ For examples, the mucin encoding gene *MUC4* and the glycosyltransferase *B3GALNT2*.^33^ Studies in mouse models of congenital disorders of glycosylation (CDGs) and behavioral phenotypes observed in CDG patients support the idea that glycogene variants either cause or contribute to the development of idiopathic ASD.^32^ Retinoids are vitamin A and its derivatives. The retinoid signaling plays a significant role in regulating brain functions, including neuronal differentiation, neurite growth, patterning of the anteroposterior axis of the neural tube, neurotransmitter release, and long term potentiation.^34^ Defective retinoid signaling has been evidenced in the pathology of Alzheimer’s disease,^35^ and Vitamin A deficiencies were reported to exacerbate symptoms in children with ASD.^36^

### SNVs correlated with bacteria taxa

**Figure 3.**
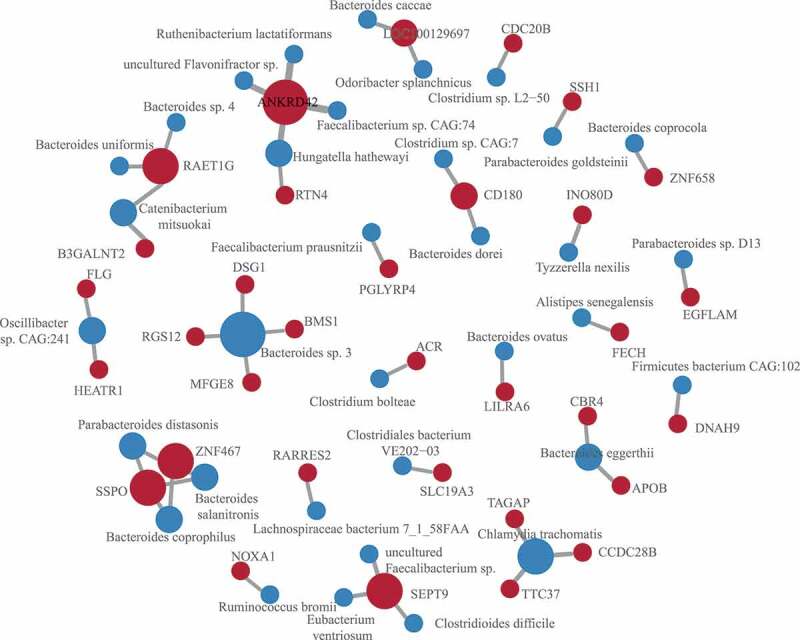
SNVs associated with microbes

Systematic screening for associations between each functional SNVs and abundance of each species was conducted using standard linear regression after adjusting for age and sex for confounding factors. There were 46 candidate associations identified (Figure 3, Table S2). Among the results, a variation in the glycosyltransferase *B3GALNT2* (chr1:235659576, c.C155T, p.A52V) was associated with the increased abundance of *Catenibacterium mitsuokai* in a subset of ASD children. A variation of *PGLYRP4* (rs148195147, c.G602A, p.R201Q) was related to *Faecalibacterium prausnitzii*. The *PGLYRP4* is a part of the innate immune system and encodes a known antibacterial protein that recognizes peptidoglycan, a ubiquitous component of bacterial cell walls. The deficiency of *PGLYRP4* leads to changes in the gut microbiota.^37^ Other associations involved by the immunity-related genes include *CD180* variation (chr5:66479217, c.C1454A, p.T485N) associated with *Bacteroides dorei* and *Clostridium sp. CAG:7, RAET1G* variation (rs4870111, c.G668A, p.R223K) associated with two Bacteroides species and *Catenibacterium mitsuokai*, and *RTN4* variation associated with *Hungatella hathewayi. CD180* is a TLR-associated molecule that is mainly expressed on macrophages, DCs, and B cells, which can also sense bacterial LPS to trigger the growth of dormant multiplemyeloma cells.^38^ Both *RAET1G* and *RTN4* mutations were reported in ASD cohorts.^22^ *RAET1G* encodes a member of the major histocompatibility complex (MHC) class I family of proteins. It is one of the ligands of natural killer group 2, member D (*NKG2D*) receptor, which functions as an activating receptor in innate and adaptive immunity.^39^ *RTN4* is necessary for immune responses triggered by nucleic acid-sensing TLRs.^40^ Besides, *RTN4* is also an inhibitor of axonal regeneration in the CNS.

SNV-microbe associations identified by regression. The SNVs were mapped to genes. The size of the node is proportional to the number of associations involved by the gene or microbe.

Also, *APOB* (Apolipoprotein B, rs13306187, c.G4163A, p.R1388H) and *RARRES2* (Retinoic Acid Receptor Responder 2, rs147597725, c.G440A, p.S147N), two genes involved in the retinoid metabolic process, were related to the abundance of *Lachnospiraceae bacterium 7_1_58FAA* and *Bacteroides eggerthii*, respectively. Apolipoprotein B was an innate barrier against bacterial infection,^41^ and novel *APOB* mutations were observed in autism.^42^ The *Bacteroides eggerthii* was reported to enhance colitis in mice.^43^ Studies have reported that the Bacteroidetes/Bacteroidales were among the key taxa related to vitamin A in ASD children.^44^ Other associations include the thiamine transporters *SLC19A3* (rs117864472, c.A1132G, p.I378V) and *Clostridiales bacterium VE202-03*, among others. The thiamine deficiency was observed in Alzheimer’s disease and contribute to synapse and neural circuit defects.^45^

**Figure 4.**
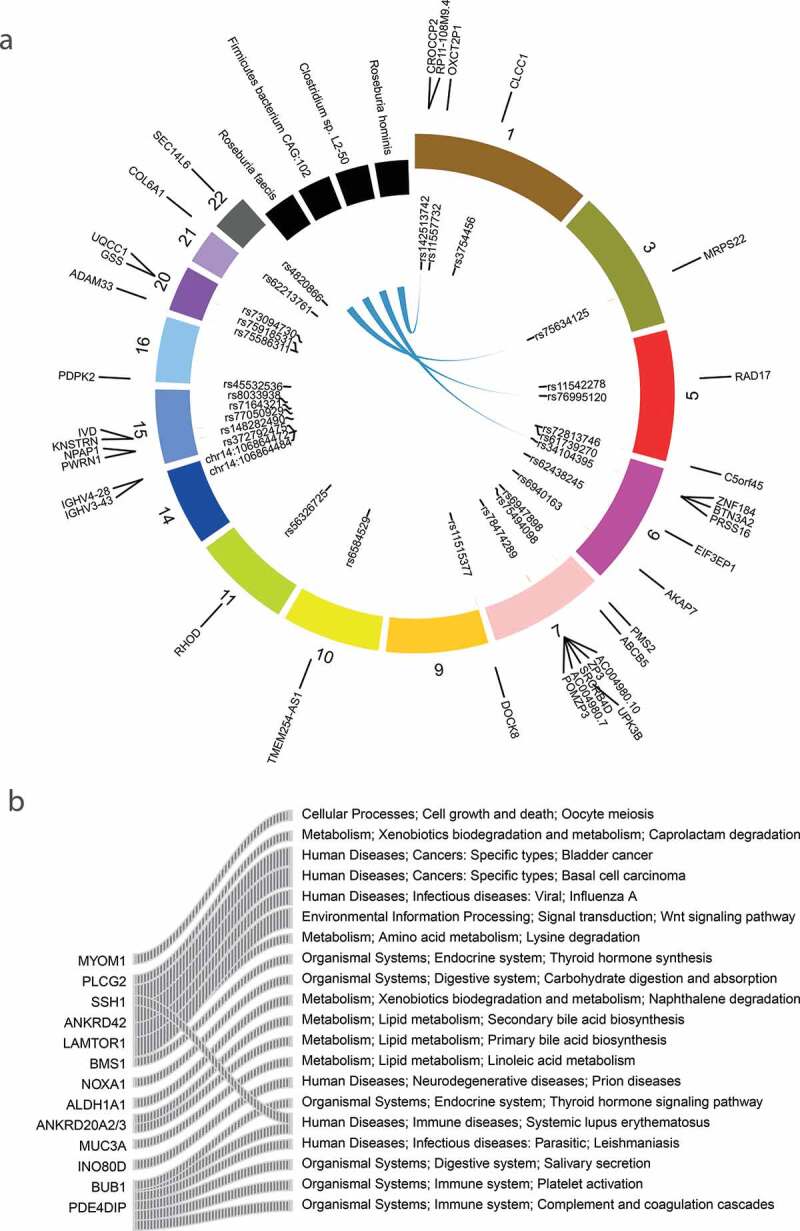
The association between QTL SNVs and microbial pathways, the causal relationship between SNVs, microbes, and metabolites

Previous studies have demonstrated that ASD genetic risk can act in part through expression quantitative trait loci,^46^ namely, eQTL, and epigenetic regulation,^47^ such as meQTL. Accordingly, we explored the association between microbes and the eQTL or meQTL SNVs. We mapped the total SNVs to the publicly available eQTL^48^ and meQTL^47^ maps for multiple tissues. Thirty SNVs in our ASD cohort were the eQTL or meQTL loci of neuropsychiatric disorders (Figure 4(a)). However, a limited number of these loci were associated with microbes (Figure 4(a)). An exonic variation in the non-coding RNA LOC285819 (rs34104395), which has an eQTL effect on *BTN3A2* in both colon and brain tissue, was associated with *Clostridium sp. L2-50. BTN3A2* is a member of the immunoglobulin superfamily, residing in the juxta-telomeric region of the major histocompatability class 1 locus. Increased expression of *BTN3A2* might confer risk for schizophrenia by altering excitatory synaptic function. Previous studies have reported the influence of MHC gene variation in shaping gut microbiome composition in both mice^49^ and human.^50^ This observation suggested a possible genetic regulation of gut microbiota mediated by the host immune system. Besides, the eQTL loci (rs75634125) of *MRPS22* (Mitochondrial Ribosomal Protein S22) was associated with the abundance of *Roseburia faecis*. The *MRPS22* mutation was reported to be associated with the mitochondrial disorder.^51^

(A) The association between eQTL/meQTL loci and microbes, and the genes putatively regulated by these loci. The inner labels are ASD SNVs that mapped to the eQTL or meQTL map, and the outermost labels represent genes regulated by the eQTL or meQTL loci. The blue links represent the associations between ASD SNVs and the gut microbes. (B) The associations between SNVs (gene) and microbial pathways (KEGG pathway, levele 3).

### Host SNVs associated with microbial pathways and microbiome gene abundance

To assess how and to what extent host SNVs impact the functions of microbiome, associations between SNVs and microbial pathways and genes were tested. We identified 22 associations between 13 host genes and 20 microbial pathways (Figure 4b, Table S3). Several variations were associated with metabolism pathways, including variations located in *MUC3A* (chr7:100550525,c.C1106T, p.P369L; chr7:100550534, c.C1115G, p.S372 C) *and ANKRD20A2/3* (Ankyrin Repeat Domain 20 Family Member A1, rs201420500, c.C2456A, p.S819Y) that are associated with lipid metabolism pathways, and *LAMTOR1* variation (rs146341570, c.C374T, p.P125L) that associated with lysine degradation. Notably, variations in *PLCG2* (rs201080992, c.C579G, p.H193Q) and *ALDH1A* (rs8187929, c.A529T, p.I177F) were associated with the pathway of Xenobiotics biodegradation and metabolism (caprolactam degradation and naphthalene degradation pathways). *ALDH1A1* is a multifunctional enzyme with dehydrogenase, esterase, and anti-oxidant activities. Moreover, coding variation in *PLGG2* implicated microglial-mediated innate immunity in Alzheimer’s disease.^52^ Toxin exposure has been epidemiologically demonstrated as one of the main etiological factors of ASD, and deficiencies in toxicant-degradation pathways were reported in ASD.^53^ These results implicated the possible genetic factors that were underlying the microbial detoxification deficiency. Besides, a couple of gene variations were associated with the immune system. For example, variation in an ASD-related gene *SSH1* (rs117900986, c.A1862G, p.N621S) was associated with the human immune disease. *SSH1* encodes proteins dephosphorylate and activate the actin-binding/depolymerizing factor cofilin. It is required by *NOD1* to detect bacterial-induced changes in actin dynamics leading to NF-κB activation and innate immune responses.^54^ Other associations were observed between variation within *NOXA1* (rs201388549, c.C526T, p.R176W), an activator of Nox2-based NADPH oxidase,^55^ and the carbohydrate digestion and absorption, and variation of *ANKRD42* (rs14346246, c.G1547A, p.G516D) with the wnt signaling pathway.

Furthermore, compared to the total annotated genes, the host-SNVs-associated genes were enriched in multiple metabolism pathways in the KEGG annotation (Table S4), including carbohydrate metabolism, amino acid metabolism, metabolism of cofactors, nucleotide metabolism and vitamins, and biosynthesis of other secondary metabolites. Additionally, enriched pathways include membrane transport (Figure S2A). Also, these microbial genes were enriched in cell motility for the eggNOG annotation (Figure S2B).

### The causal relationship between SNV, microbiome and its metabolites

**Figure 5.**
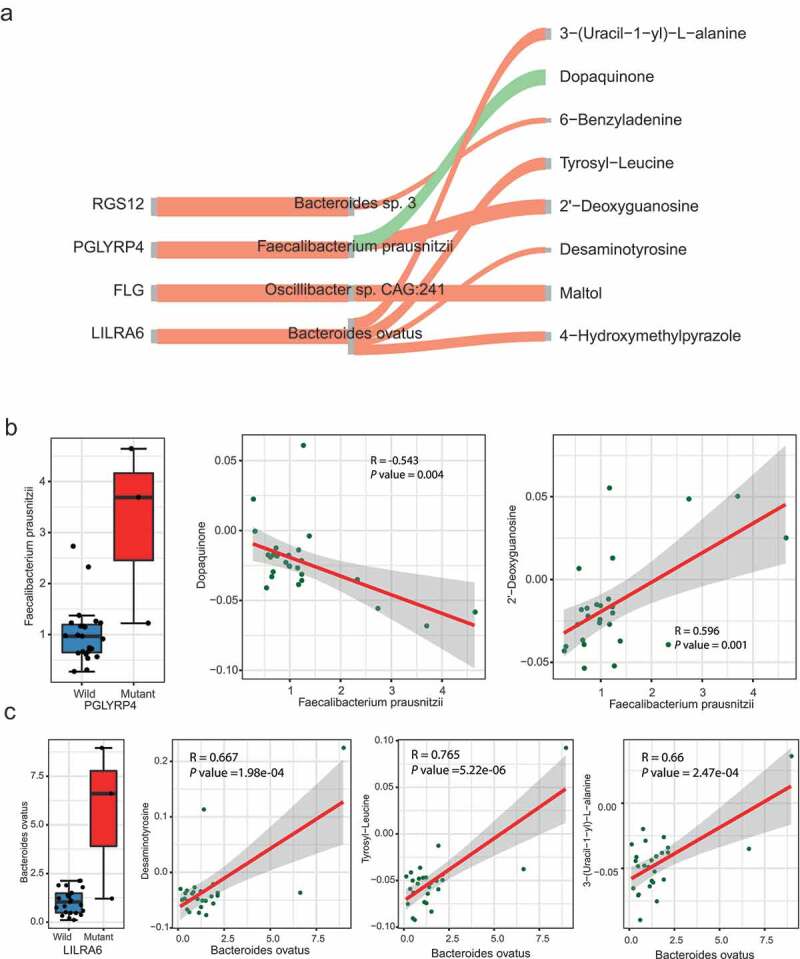
The causal relationship between SNVs, microbes, and metabolites

One of the critical mechanisms by which microbes regulate host physiology is through their metabolites.^15,56^ To decipher how the host SNVs impact microbiome and their metabolites in ASD, a causal inference test (CIT) proposed by Millstein et al.^57^ was applied to infer the causal relationship between SNV, microbes and their metabolites. Focusing on the SNVs associated with specific microbes identified in the previous section, 8 SNV-microbe-metabolite trios, comprising 4 SNV-microbe associations detected before and 8 metabolites (Figure 5(a), Table S5), were identified.

(A) The causal relationship of SNV-microbe-metabolite trios inferred by causal inference test. The red and green links represent positive and negative assoications, respectively.(B) The abundance of *Faecalibacterium prausnitzi* related to *PGLYRP4*a SNV status, and the correlation between *Faecalibacterium prausnitzi* and Dopaquinone and 2ʹ-Deoxyguanosine.(C) The abundance of *Bacteroides ovatus* related to SNV status of *LILRA6* and the correlation between *Bacteroides ovatus* and Desaminotyrosine, Tyrosyl-Leucine, and 3-(Uracil-1- yl)-L- alanine.

Three metabolites in the putative causal relationship network were involved in tryptophan and tyrosine metabolism, i.e. Tyrosyl-Leucine, Dopaquinone, and Desaminotyrosine. The level of Dopaquinone was causally related to the variation of *PGLYRP4* (rs148195147, c.G602A, p.R201Q) and the abundance of *Faecalibacterium prausnitzii* (Figure 5(b)). Also, the *Faecalibacterium prausnitzii* is associated with the level of the 2ʹ-Deoxyguanosine, a metabolite involved in the folate biosynthesis pathway. A variation located in *LILRA6* (rs56257556, c.A931 G, p.N311D) was associated with Desaminotyrosine, Tyrosyl-Leucine, and an Alanine metabolites 3-(Uracil-1-yl)-L-alanine mediated by *Bacteroides ovatus* (Figure 5(c)). *LILRA6* is a leukocyte immunoglobulin-like receptor and plays roles in the pathways of the innate immune system and class I MHC mediated antigen processing and presentation. The *de novo* mutation of *LILRA6* was previously observed in ASD.^58^

### Association of cytokines with host genetics and microbiota

**Figure 6.**
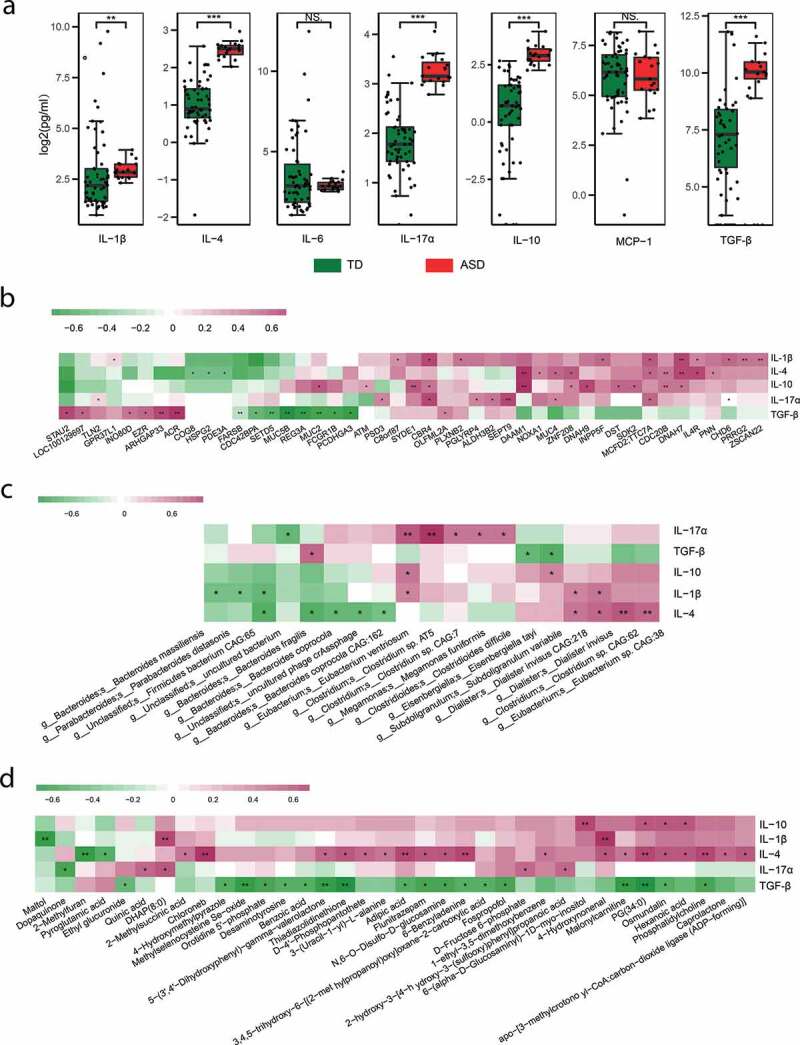
Cytokines in ASD and their association with gene, microbes, and metabolites

Emerging research suggests that immune dysfunction is a risk factor contributing to the neurodevelopmental defects observed in ASD.^59^ We then tested the levels of seven cytokines in the blood serum (Table S6). The level of IL-1β, IL-4, IL-17α, IL-10, and TGF-β was significantly higher in ASD children compared with TDs (Figure 6(a)), consistent with the previous studies.^8,17,18^ Several studies have suggested that host genetic can reshape the gut microbiome and its metabolites through aberrant immune activation.^8^ The association between genetic variation and the differing cytokines was tested. The level of all the five differentially expressed cytokines was associated with SNVs (Figure 6(b), Table S7). Including known ASD-associated genes *MUC2, MUC4, SETD5, MUC5B, INO80D, SEPT9, PGLYRP4, DST*, and *CHD6*, among others. Besides, all the five cytokines were correlated with microbes and metabolites (Figure 6(c-d), Table S8-9). These results indicated that the interaction of gut microbes-metabolites and host genetics might play essential roles in the aberrant immune activation in ASD children.

(A) The level of in cytokines in ASD and control samples, *Wilcoxon test*, ***fdr<0.001, **fdr<0.01, and *fdr<0.05.(B) The heatmap of associations between SNV (gene) and cytokines, * linear regression, *p-value*<0.05, ** *p-value*<0.01.(C) The heatmap of associations between microbes and cytokines, Spearman correlation, * *p-value*<0.05, ** *p-value*<0.01.(d) The heatmap of associations between metabolites and cytokines, Spearman correlation, * *p-value*<0.05, ** *p-value*<0.01.

## Discussion

**Figure 7.**
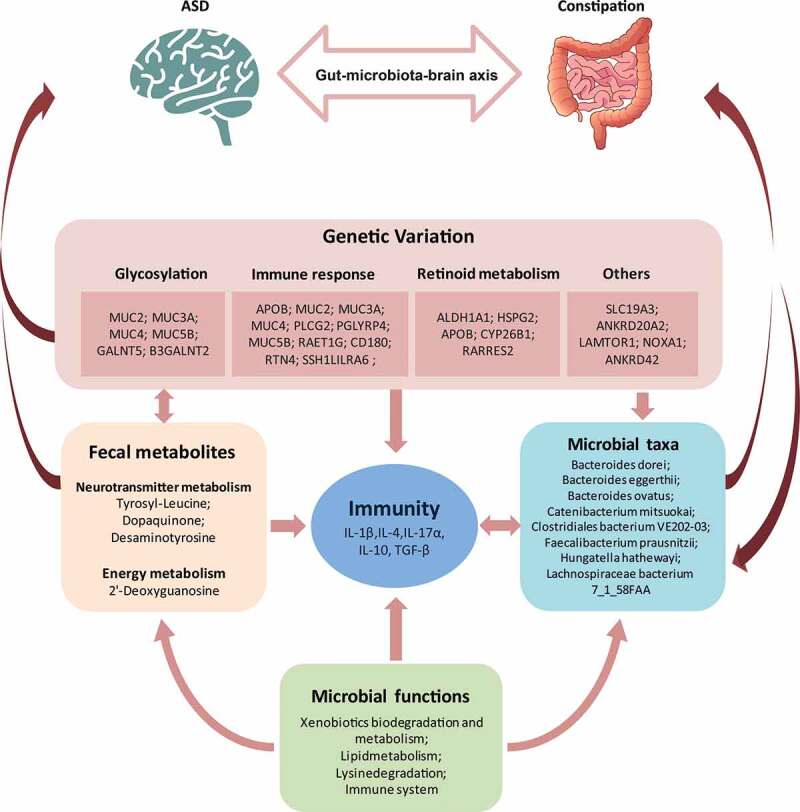
The summary of the interplay among host genetics, gut microbes, fecal metabolites, and cytokines

In this study, we first investigated the interplay between host and gut microbial in terms of host genetic, microbial composition, microbial gene abundance, microbial metabolites, and host immunity in ASD. We provided new insight into the interaction between host genetic variations and gut microbiota in the pathogenesis of ASD (Figure 7).

The link between ASD and the gut microbiome has been highlighted by many recent studies.^12,15,60,^^61,62^ Given that ASD is a disease influenced by both genetic and environmental factors, it is of interest to study the microbiome in the context of host genetic variation. Here, we jointly analyzed the SNV profile and the taxonomic composition of the fecal microbiome as well as its metabolites, and additionally, the host cytokines in ASD. We found that the composition of the microbiome is correlated with functional SNVs. The functional SNVs located in genes involved in glycosylation process, innate immune response, and retinoid metabolic process were significantly enriched in the presence of ASD, indicating that the abnormal function of proteins that participated in these biological processes may be involved in the pathogenesis of ASD. Consistent with that, the subjects recruited in this study all had symptoms of constipation, the SNVs identified in the samples were enriched in the genes associated with gut functions, e.g. the mucin gene (*MUC2, MUC3A, MUC4*, and *MUC5B*). The mucins can act as the protective barrier or luminal sensor for gut immunity and regulate gut microbiota.^63^ Also, the increased gastric mucin was observed in the treatment of chronic constipation with Lubiprostone.^64^ More importantly, the mucin gene mutation was reported in multiple ASD cohorts.^42,65,66^ The association analysis between functional SNVs with microbes in this current study revealed the involvement of SNVs within MUC family genes in the interaction between gut microbiota of constipated ASD.

Immune dysfunction is a viable risk factor contributing to the neurodevelopmental deficits observed in ASD,^59^ and many autism susceptibility genes are related to immune/infection pathways.^67^ On the other hand, microbes influence the activation of peripheral immune cells and contributes to the etiopathogenesis of neurobehavioral and neurodegenerative disorders, such as ASD, anxiety depression, Alzheimer’s disease and Parkinson’s disease.^68^ In this study, we found several associations between the immune-related gene variations and gut microbial taxa. For example, the variations located in *PGLYRP4, APOB, CD180, RAET1G*, and *LILRA6* were associated with multiple species, mainly the *Bacteroides*, altered abundance of which was reported in multiple ASD studies.^69^ These observations implicated a scenario where the host genetic shaped the gut microbes and indicated an interplay between immunity and microbes in the context of ASD. However, for the other type of SNVs (intergenic, upstream/downstream, intronic SNVs, and non-coding exonic SNVs), thought tens of them were mapped to eQTL or meQTL loci, a limited number of these SNVs were associated with the abundance of microbes (Figure 4(a)). There were two possibilities regarding this observation. First, the interplay between genetic variations and gut microbiota abundance in ASD might occur mostly through the loss-of-function (LoF) gene variations. Second, another possible explanation is that due to the small sample size of our study, there was not enough statistical power to detect indirect associations between SNVs and microbes, which were mediated by the eQTL or meQTL genes. In this case, by increasing the sample size, we may expect an increased number of associations between nonfunctional SNVs and gut microbes being observed.

In the association analysis between host SNVs and the gut microbial pathways and genes, we observed notable associations between the SNVs and immune system and metabolic pathways. Metabolites act as messengers of information between the intestinal microbiota and host cells. The presence of metabolites, which depends on the microbial metabolic activity, thus impacts host development, health, and pathogenesis.^70^ For example, amino acids serve as precursors for many neuroactive molecules, such as serotonin, and GABA. The amino acid metabolism were reported to be different between TD and ASD individuals.^71,72^ The association between host SNVs with microbial genes related to amino acid metabolism suggested a mechanism through which ASD host genetic variations influence neurodevelopment and behavior in a microbe-mediated way. The additional causal inference test also identified multiple genetic variations associated with the level of metabolites involved in the neurotransmitter metabolism. This result provides a possible mechanism underlying the genetic susceptibility of ASD. To identify variations with higher accuracy, we only considered variations detected in more than two samples. Considering the small sample size of our study, some rare or less common variations which may also contribute to the pathogenesis of ASD will be filtered. Thus, it is necessary to recruit more patients to capture such variations linking to ASD.

Taken together, the current study provides important clues to explore the mechanism of ASD from the perspective of genetic and environmental interaction. Many studies describe gut dysbiosis, disorders of metabolism activity, and immune dysfunction, as a change in ASD. However, little is known about whether these changes are a cause or consequence of an altered pathological state. Therefore, further animal experiments are needed to understand exactly how the candidate genetic variants influence the interplay between microbiota, metabolism, and the immune system in pathology of ASD.

## Materials and methods

### Ethics statement

This study was approved by the Ethics Committee of Affiliated Yixing Hospital of Jiangsu University (Ethics NO, 2016055). All participants and their legal guardians were provided a written informed consent upon enrollment. Once the consent forms were signed, we screened them for eligibility criteria and sent questionnaires and sample collection kits to participants who meet the study eligibility criteria.

### Study subject recruitment

The children with ASD were recruited as we described in our previous study^73^. Briefly, ASD children were diagnosed according to the Diagnostic and Statistical Manual of Mental Disorders, 5th Edition. Each participant underwent physical, neurological, and behavioral examinations. Children were excluded from the study if they were previously diagnosed with a genetic condition such as tuberous sclerosis, significant sensory impairment, clinically evident inflammatory conditions, celiac disease, and other physiological conditions (i.e. depressive disorder, schizophrenia, and bipolar disorder). Participants that are on anti-inflammatory or anti-oxidant drugs, or take antibiotics, probiotics, prebiotics, and antifungal medications in 3 months before the feces collection were excluded. The Rome IV criteria for functional constipation were used for evaluating GI problems. The typically developed children (TD) for the fecal metabolism and cytokines measure were recruited from kindergartens through pediatricians and under physical, neurological, and behavioral examinations as the ASD group, subjects with any pathological conditions, including GI problems were excluded (Table S10).

### Exon sequencing and data process

Peripheral blood samples were collected from the ASD and TD group for extracting DNA. Exome capture was performed using the Agilent SureSelect Human All Exon V6 r2 kit followed by Illumina paired-end sequencing.

The raw reads were quality-controlled using FastQC v0.11.2 and then mapped to the human reference genome (hg19) using bwa v0.7.17.^74^ PCR duplicates were removed using Picard tools v2.18.12. SNVs were identified using FreeBayes v1.2.0^75^ and filtered using bcftools v1.9. SNVs with base quality < 30, coverage < 30, with less than 3 reads mapped to the alternative alleles or located within 5bp nearby a gap were removed.

SNVs presented in the TD groups were removed. Then, SNVs were annotated by the annovar software to extract allele frequencies from the public database Allele frequency of these SNVs in the normal population was extracted from the 1000 Genome project,^76^ the Exome Aggregation Consortium (ExAC) database,^77^ and the Chines MillionomeI database (CMDB),^78^ a large-scale Chinese genomics database including comprehensive variation and their allele frequency information from 141,431 unrelated healthy Chinese individuals. SNVs with MAF < 5% in these public databases and detected in more than two ASD samples were considered as candidates. GO enrichment analysis of genes with SNVs was conducted in R package.

### Fecal sample collection

Fecal samples of each participant were obtained at the hospital or home and collected as directed in the instruction provided and frozen immediately until shipment. Samples were shipped using dry ice overnight to Nanjing Medical University and stored at −80°C until extraction.

### Metagenomic sequencing and preprocess

About 2 μg DNA per sample was prepared for library construction and then sequenced on the Illumina HiseqX platform. Low quality or contaminated human reads were removed, and the high-quality reads were assembled into contigs using SOAPdenovo (v2.04).^79^ MetaGeneMark (v2.10)^80^ was used to predict genes from the assembled contigs. CD-HIT v4.5.8^81^ was used to generate a non-redundant gene catalog. Abundances of the genes were computed by aligning high-quality reads to the reference gene database. For taxonomic identity and functional assignment of unigenes, reads were aligned to the NCBI NR database (e-value≤1e-5) using DIAMOND (v0.9.9).^82^ The LCA algorithm^83^ was used to conduct annotation. The detailed methods and results have been presented by our other study.

### Gene functional annotations

The unigenes were functionally annotated by mapping to different functional protein databases with BLAST software. Predicted unigenes were assigned to the Kyoto encyclopedia of genes and genomes (KEGG), the evolutionary genealogy of genes: non-supervised orthologous groups (eggNOG), and carbohydrate-active enzymes database (CAZy) database using DIAMOND (v0.9.9). The abundances of each functional annotation were the sum of the abundance of annotation of each functional level.

### Metabonomics analysis and data process

For each ASD and TDs, about 50 mg feces were mixed with 1000 μL of extract solvent, and samples were transferred to UHPLC-QE Orbitrap/MS analysis. The differential analysis was performed using the ropls package(v1.12.0), and metabolites were with VIP > 1, *P*-value (T-test) < 0.05 and FC>1.5 were defined as significant different in abundance. The detailed methods have been presented in our previous study.

### Association analysis

Association test was performed using standard linear regression after adjusting for established confounding factors (age and sex). *p-values* were then corrected for multiple tests using the Benjamini-Hochberg method.

### Causal inference analysis

The SNV-microbe-metabolite trios were assessed using the causal inference test (CIT)^57^ to test the possibility that SNV causally influences microbe abundance and then the microbe metabolite. The enriched SNVs and their associated microbes, and the differential metabolites between ASD and TD group were subjected to the test. Briefly, the CIT has statistical tests for four conditions, all of which must be met for the microbe -mediated causal classification: (1) SNV and microbe are associated; (2) SNV is associated with microbe after adjusting for metabolite; (3) microbe is associated with metabolite after adjusting for SNV; and (4) SNV is independent of metabolite after adjusting for microbe. The CIT *p-value* was defined as the maximum of the component test *p-values*, and multivariate linear regression was used in the four-component tests.

### Cytokine assay in healthy control and ASD patients

We measured cytokines in blood serum of the ASD cohort and an independent TD group including IL-1ß, IL-4, IL-6, IL-10, IL-17α, MCP-1, and TCG-β using BD™ CBA Flex Set (Table S6). Then the level of them was log-transformed, and the Mann-Whitney U test was applied to test the difference between ASD and TDs. These *p-values* were then corrected for multiple tests using the Benjamini-Hochberg method and cytokines with *FDR*<0.05 were considered as differentially expressed.

## Supplementary Material

Supplemental Material

## Data Availability

All metagenomic raw data have been deposited in GEO under accession number GSE113540.
