# Supplementary material for: Gene variations in Autism Spectrum Disorder are associated with alternation of gut microbiota, metabolites and cytokines
Source: Gut Microbes. 2021 Jan 8;13(1):1854967. doi: 10.1080/19490976.2020.1854967 (PMC7808426; doi:10.1080/19490976.2020.1854967)
Supplement: Supplemental Material [file KGMI_A_1854967_SM8160.zip › Supplementary files/Figure 1.pdf]

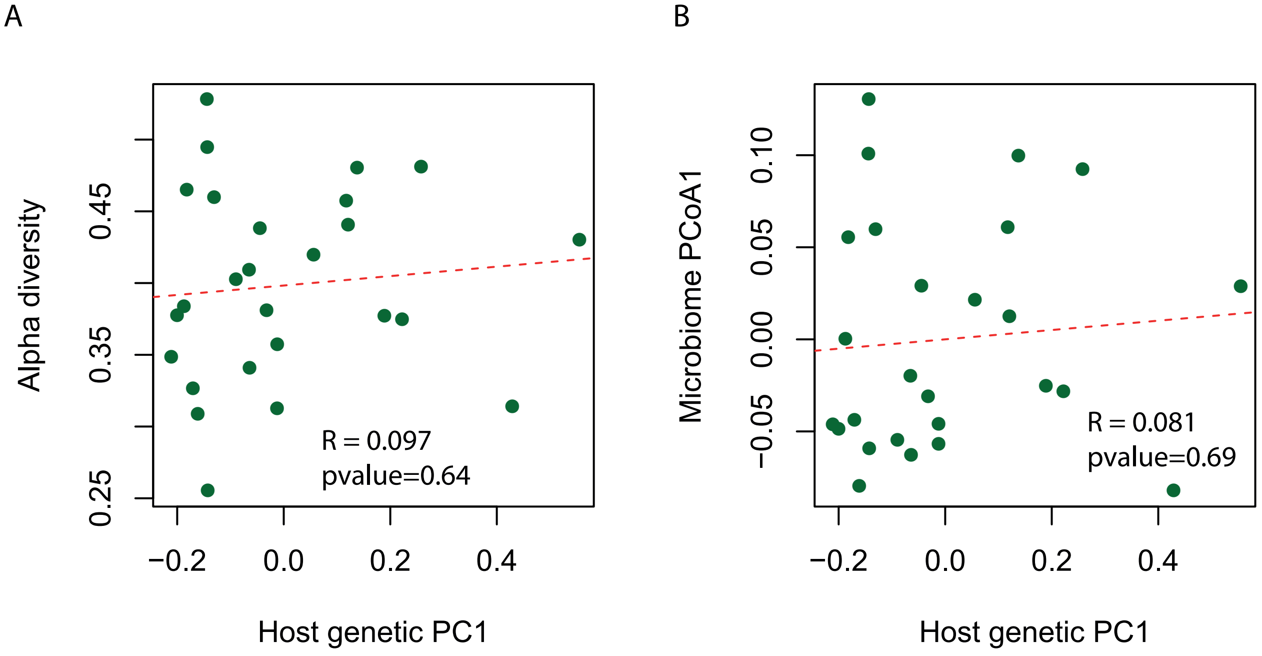

Figure S1. The correlation between total SNVs enriched in ASD with microbiome diversity (A) and composition (B)
