# Supplementary material for: Gene variations in Autism Spectrum Disorder are associated with alternation of gut microbiota, metabolites and cytokines
Source: Gut Microbes. 2021 Jan 8;13(1):1854967. doi: 10.1080/19490976.2020.1854967 (PMC7808426; doi:10.1080/19490976.2020.1854967)
Supplement: Supplemental Material [file KGMI_A_1854967_SM8160.zip › Supplementary files/Figure 2.pdf]

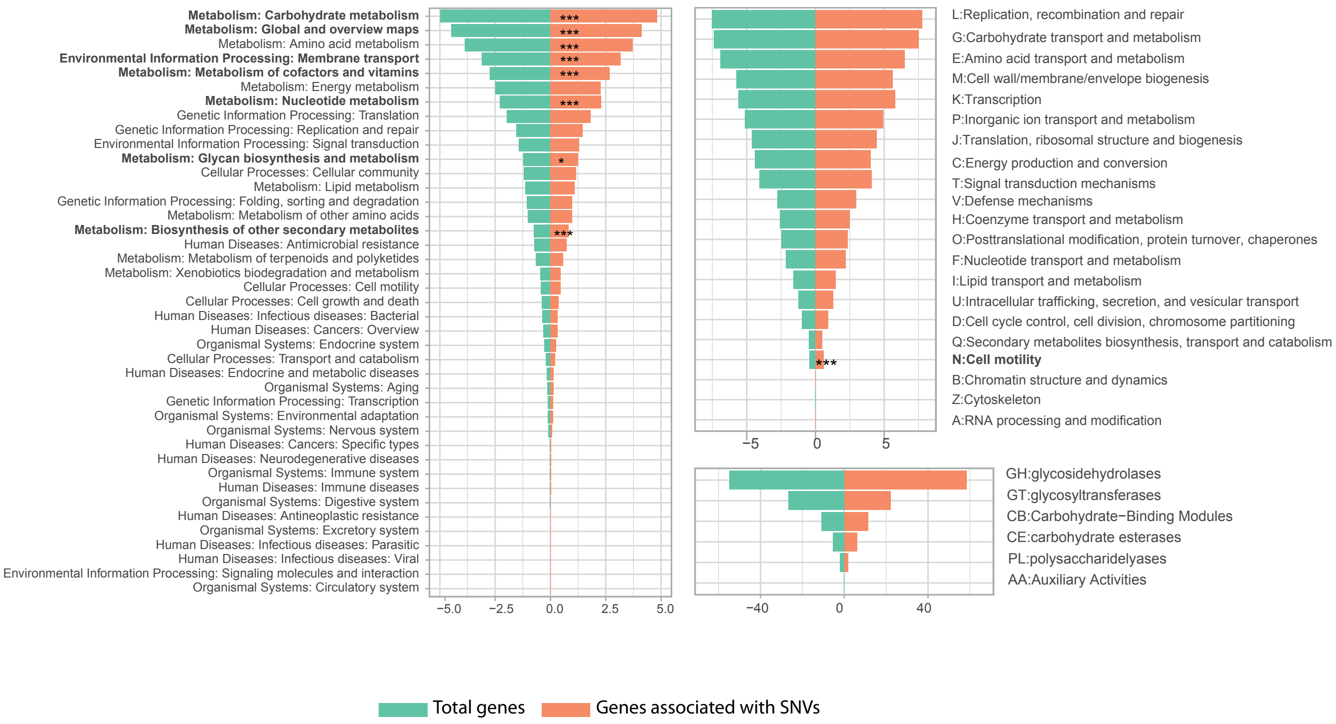

Figure. S2. Functional enrichment of SNV associated metagenomic genes. (A-C) The percentage of total metagenomic genes (orange) and metagenomic genes whose abundance were significantly associated with host SNVS(green) annotated to each functional terms based on. A hypergeometric test was used to test the enrichment of SNV-associated genes in each functional terms: ***fdr<0.001, ** fdr<0.01 and * fdr<0.05.
